# Purinergic Signaling and Related Biomarkers in Depression

**DOI:** 10.3390/brainsci10030160

**Published:** 2020-03-12

**Authors:** Francesco Bartoli, Geoffrey Burnstock, Cristina Crocamo, Giuseppe Carrà

**Affiliations:** 1 Department of Medicine and Surgery, University of Milano Bicocca, Via Cadore 48, 20900 Monza, Italy; cristina.crocamo@unimib.it (C.C.); giuseppe.carra@unimib.it (G.C.); 2 Department of Mental Health & Addiction, ASST Nord Milano, Bassini Hospital, via Gorki 50, 20092 Cinisello Balsamo, Milano, Italy; 3 Department of Pharmacology and Therapeutics, University of Melbourne, Parkville, Victoria 3010, Australia; g.burnstock@ucl.ac.uk; 4 Division of Psychiatry, University College London, London, Division of Psychiatry, University College London, 6thFloor, Maple House, 149 Tottenham Court Road, London W1T7NF, UK

**Keywords:** purinergic system, adenosine, ATP, caffeine, biomarkers, depression, molecular psychiatry

## Abstract

It is established that purinergic signaling can shape a wide range of physiological functions, including neurotransmission and neuromodulation. The purinergic system may play a role in the pathophysiology of mood disorders, influencing neurotransmitter systems and hormonal pathways of the hypothalamic-pituitary-adrenal axis. Treatment with mood stabilizers and antidepressants can lead to changes in purinergic signaling. In this overview, we describe the biological background on the possible link between the purinergic system and depression, possibly involving changes in adenosine- and ATP-mediated signaling at P1 and P2 receptors, respectively. Furthermore, evidence on the possible antidepressive effects of non-selective adenosine antagonist caffeine and other purinergic modulators is reviewed. In particular, A2A and P2X7 receptors have been identified as potential targets for depression treatment. Preclinical studies highlight that both selective A2A and P2X7 antagonists may have antidepressant effects and potentiate responses to antidepressant treatments. Consistently, recent studies feature the possible role of the purinergic system peripheral metabolites as possible biomarkers of depression. In particular, variations of serum uric acid, as the end product of purinergic metabolism, have been found in depression. Although several open questions remain, the purinergic system represents a promising research area for insights into the molecular basis of depression.

## 1. Introduction

The purine nucleoside adenosine was identified in 1929 by Drury and Szent-György [1], who described the physiological activity of adenine compounds in the mammalian heart. The hypothesis that adenosine-5’-triphosphate (ATP) and related nucleotides might function as neurotransmitters was postulated during the 1970s [2,3]. It is now established that ATP and adenosine can influence a wide range of physiological functions, including neurotransmission and neuromodulation [4,5]. Purinergic receptors were differentiated into two families, P1 and P2 receptors, activated by adenosine and ATP, respectively [6,7]. Extracellular adenosine and ATP levels are determined by the balance between the metabolic action of ectonucleotidases and the release from cells. The ectonucleoside triphosphate diphosphohydrolase-1, also known as CD39, converts ATP and adenosine-diphosphate (ADP) into adenosine-monophosphate (AMP). On the other hand, the ecto-5′-nucleotidase, also known as CD73, converts AMP to adenosine [8]. Adenosine can be released in the extracellular space via equilibrative nucleoside transporters (ENTs) [9]. Under physiological conditions the release of ATP from astrocytes, followed by the degradation into adenosine via ectonucleotidases, has been identified as a major source of synaptic adenosine [10]. Adenosinergic signaling via the G-protein coupled P1 receptors (A1, A2A, A2B, and A3 subtypes) [6,7,11,12] has a role in neurodevelopmental and pathophysiological processes, such as inflammation, cell proliferation, differentiation, and neuron–glia crosstalking [13]. The impact of adenosine on brain function is mainly dependent on the activity of A1 and A2A receptors, while limited action on central nervous system (CNS) functions has been shown for A2B and A3 receptors [14]. A1 receptors are the most abundant and homogenously distributed in the brain [7], with high expression levels in the cerebellum, hippocampus, cortex, and thalamus, whereas A2A receptors are highly expressed in striatopallidal neurons, with a lower presence in other brain regions [15,16]. The primary function of adenosine seems to be inhibitory neuromodulation, linked with a negative feedback to excitatory activity of glutamatergic synapses [17]. Presynaptic A1 receptors inhibit the release of neurotransmitters, including glutamate, dopamine, serotonin, and acetylcholine, while postsynaptic receptors reduce neuronal signaling by hyperpolarization and excitability via regulation of potassium channels [16]. A2A receptors may enable adaptive responses in the regulation of synaptic plasticity. The adenosinergic system as a whole promotes pre- and post-synaptic modulatory effects on neurotransmission and is involved in synaptic plasticity and neuroprotection [14]. The activity of ATP is mediated by P2 receptors, which were further divided into two subtypes, i.e., ionotropic P2X and metabotropic P2Y receptors [18,19]. Sources for extracellular ATP in the nervous system may include neurons, glia, endothelium, and blood [20]. Extracellular ATP contributes to neurotransmission and neuromodulation, as well as to the regulation of microglia and astrocyte activities [19]. Dysfunctions of purinergic signaling, at a genetic, biochemical, or functional level, may lead to altered behaviors and mood abnormalities [21]. In particular, the purinergic system may play a role in the pathophysiology of major depressive disorder, influencing neurotransmitter systems and hormone pathways of the hypothalamic-pituitary-adrenal axis [13]. Components of purinergic signaling and related metabolism of adenosine may be implicated in depressive disorders. 

In this overview, we describe the biological background of the possible link between the purinergic system and depression, summarizing epidemiological and preclinical evidence of the possible effects of caffeine and other purinergic modulators, as well as the role of relevant biomarkers in depression. Ultimately, this may help in clarifying the possible involvement of the purinergic system in major depressive disorder.

## 2. The Adenosine Receptor Antagonist, Caffeine, and Depression

The possible role of the purinergic system and, in particular, of adenosine and P1 receptors in depression is mainly derived from studies on the association between caffeine consumption and related mood changes [22,23]. P1 receptors are antagonized by methylxanthines and their derivatives, including caffeine (1,3,7-trimethylxanthine), which is a non-selective antagonist of A1 and A2A receptors [11,24]. Caffeine studies provided insights into the possible effects of adenosine, including the potential influence on mental health [25]. Moderate doses of caffeine may improve anxious and depressive symptoms, whereas excessive doses may induce anxious, stimulant, and ‘mania’-like symptoms [25]. A meta-analysis based on 11 observational studies showed protective effects of caffeine on depression, with relevant risk decreasing by 8% for each cup/day increment in coffee intake [26]. Consistently, a wider meta-analysis showed that consumption of coffee and, partially, of tea might decrease the risk of depression [27]. However, dose-response effects suggested a nonlinear J-shaped relationship, with a peak of protective effect for 400 mL/day of caffeine. Additionally, results from three large US cohorts estimated an association between higher caffeine consumption and lower risk of suicide. The relative risk for suicide was 0.75 (0.63–0.90) for each increment of 2 cups/day of caffeinated coffee and 0.77 (0.63–0.93) for each increment of 300 mg/day of caffeine [28]. Finally, a large cohort study conducted in Korea on 80,173 individuals showed that regular and moderate caffeine intake was likely to reduce suicide risk and depression in women, despite higher consumption levels associated to worse outcomes [29]. This study confirmed findings of previous epidemiological data showing again a J-shaped association of caffeine with the risk of suicide [30].

## 3. Adenosine and Depression

Based on the potential effects on depression attributable to the non-selective A1 and A2A receptor antagonist caffeine, the role of adenosine in depression has attracted attention. It has been shown that fluoxetine and nortriptyline may affect the ectonucleotidase pathway in synaptosomes, suggesting that antidepressants could modulate the extracellular adenosine levels, which would result in increased adenosine in cerebral cortex and decreased in hippocampus [31]. On the other hand, it seems that chronic treatment with mood stabilizers, such as lithium, used for bipolar disorder and mania, can modulate the ectonucleotidase pathway in hippocampal synaptosomes, with a related decrease of ATP and increase of adenosine levels [32]. A1 and A2A receptors have complementary effects and a release of neurotransmitters seems dependent upon the balance between A1 and A2A receptors [12,13]. A non-selective activation of adenosine receptors seems to induce depressive-like symptoms in animal models, whereas selective antagonism of A2A receptors may induce antidepressant effects [25]. It has been shown that enhanced neuronal expression of A1 receptors led to pronounced acute and chronic resilience against depressive-like behaviors, while A1 receptor knockout mice showed increased depressive-like behaviors and resistance to antidepressant treatments [33]. On the other hand, male rats overexpressing A2A receptors exhibit depressive-like behaviors [34,35,36]. In addition, a genetic deletion of A2A receptors may prevent chronic stress-induced behavioral, neurochemical, and electrophysiological alterations in the hippocampus [37]. Rial and colleagues [38] hypothesized that depression may be associated with an astrocytic hypofunction, causing a decreased activation of inhibitory adenosine A1 receptors in neurons and, in parallel, an upregulation of synaptic adenosine A2A receptors, which is associated with aberrant plasticity. Consistently, selective A2A antagonists have attracted attention for their possible role in the treatment of depression. Preclinical studies highlighted that A2A antagonists have antidepressant effects [39,40,41,42] and may potentiate responses to antidepressant treatment [43], whereas A1 antagonists do not [44]. The A2A selective antagonist istradefylline (KW6002), recently approved by the FDA as an add-on therapy for off episodes in adults with Parkinson’s disease [45], may also be effective in treating depressive-like symptoms, with an effect that is independent from monoaminergic transmission in the brain [46]. The co-administration of istradefylline with antidepressant agents, including selective serotonin reuptake inhibitors (paroxetine or fluoxetine) or monoamine oxidase B inhibitors (deprenyl), resulted in a significant reduction of depressive-like behaviors [47]. However, the adenosinergic system is complex, involving the modulation of different neurotransmitters, and neurobiological mechanisms supporting the efficacy of A2A receptor antagonism in depression are not fully understood. Hippocampal release of serotonin, one of the major neurotransmitters implicated in depression, seems decreased by the activation of A1 receptors and increased by A2 receptor activation [13,48]. Chronic stress seems to significantly reduce adenosine levels, which, at low concentrations, may activate A1 receptors, leading to a decrease of serotonin concentration in the hypothalamus [12]. It is likely that antidepressant effects may be only partially due to the influence on serotoninergic transmission in the brain, and explained by the modulation of other neurotransmitters [14]. Antidepressive effects of selective A2A antagonists may be linked to relevant interactions with dopaminergic transmission [40]. Adenosine receptor antagonists may be able to reverse symptoms such as anergia, fatigue, and psychomotor slowing, induced by dopamine antagonism or depletion [24]. Additional mechanisms, including the possible involvement of A2A receptors in metabolism and neuroinflammation and the role of neurochemical mediators of antidepressant responses, have been considered [22]. For example, it has been shown that fluoxetine-induced upregulation of the Brain-Derived Neurotrophic Factor (BDNF), involved in depression pathophysiology [49], may be mediated by both P1 and P2 receptor signaling [50]. 

It is worth mentioning that non-pharmacological strategies for depression may also influence adenosinergic signaling [22]. Both electroconvulsive therapy and sleep deprivation are likely to induce short- and long-term adaptations of the adenosine neuromodulation system [22]. In particular, a key role of A1 receptors in determining the beneficial effects of sleep deprivation on depressive-like behaviors has been shown. Both knocking out of A1 receptors and central delivery of A1 receptor antagonist support the hypothesis of the importance of adenosinergic signaling for sleep deprivation antidepressant effects [51].

Finally, possible interconnections between adenosine receptors and suicide-related behaviors, often occurring in depression, have been hypothesized, although there is no direct evidence purposively exploring this link. It has been shown that impulsive behaviors might be driven by the inhibition of A2A receptors, accompanied by an increased neuroblast proliferation in the hippocampus [52]. Thus, opposite effects of A2A receptors and related adenosine metabolism have been hypothesized to explain depression-related suicidal ideation and impulsive suicide attempts, respectively [53]. 

## 4. ATP and Depression

Along with adenosine, ATP signaling through P2 receptors may play an important role in the neuropathological mechanisms of depression [54,55,56]. An early study showed that erythrocyte membrane ATP activity was significantly lower during the depressive phase of patients than in the remission phase [57], suggesting that ATP may be involved in depression. The combination of non-specific P2 receptor antagonists with antidepressants has been associated with significant antidepressant-like effects in animal models [58]. It has been hypothesized that ATP released from astrocytes might trigger the development of depressive-like behaviors [59,60]. ATP-mediated signaling through the P2X7 receptor subtype seems to play an important role in depression [54,55,56]. The P2X7 receptor is a ligand-gated cation channel localized in different CNS cells involved in the modulation of different neurotransmitters [55]. Activation of purinergic P2X7 receptors may be involved in the pathogenesis of depression [61], possibly linked with its proinflammatory activity [62]. It has been hypothesized that psychological stress may influence the immune system in the CNS, via the ATP/P27X receptor pathway [63]. Genetic deletion of P2X7 receptors has been associated with antidepressant effects. Preclinical studies showed that P2X7 receptor knockout mice exhibited an antidepressant-like profile and higher responsivity to the antidepressant treatment [64,65]. The antidepressant phenotype related to genetic deletion of P2X7 receptors seems associated with changes in hippocampal monoaminergic transmission [66]. All these findings support the hypothesis of CNS-penetrable ATP-sensitive P2X7 receptor antagonists as novel antidepressant agents [58,59,67]. Moreover, ATP-sensitive potassium channels have been claimed to be a possible target for the treatment of depression [68,69,70]. 

## 5. Genetic Studies

A large number of studies have shown that common genetic variants of adenosine receptors may have a role in mental disorders [71]. In particular for major depressive disorders, a pilot study has shown that A1 receptor availability in several brain regions involved in emotion and mood regulation, such as the superior frontal gyrus, the dorsolateral prefrontal cortex, the hippocampus, and the entorhinal cortex, might be particularly prone to A2A polymorphism effects [72]. Recent research [73], based on 1253 individuals from a cross-sectional population-based study, examined the association between a single nucleotide polymorphism in the A2A receptor gene (rs2298383 SNP) and depression. A TT genotype was associated with a decreased likelihood of depression as compared with the CC/CT genotypes, after adjusting for several variables, including gender, smoking, socio-economic status, and ethnicity. Moreover, the TT genotype was shown to be independently associated with reduced sleep disturbances and lesser difficulty in concentrating. Contribution of adenosine related genes to the risk of depression and related sleep disturbances, was analyzed in the Health 2000 Study based on 1423 adults from the Finnish population [74]. Selecting 117 single nucleotide polymorphisms from 13 genes, a negative association between SLC29A3 polymorphism rs12256138 and depressive disorders was found among women. The results suggested that abnormalities in adenosine transport due to variation among women of the nucleoside transporter gene SLC29A3, could predispose to depression, with genetics of mood regulation possibly varying between the sexes. 

Similarly, a possible association of P2X7 gene polymorphisms with depression symptoms has been discussed [75]. Mixed results are available from previous studies, testing P2X7 polymorphisms in depression [76,77]. It seems that P2X7 receptor gene variants significantly increase the risk of mood disorders [78]. A recent meta-analysis showed a significant association between the P2X7 polymorphism rs2230912 and mood disorders (major depressive and bipolar disorders), despite pointing out the need for further studies to strengthen the evidence and clarify the applicability of the findings for pharmacological purposes [79].

Genetic polymorphisms of purinergic receptors do not seem specific to major depressive disorders and have been extensively studied in anxiety disorders and related symptoms [22]. These are likely to produce a high variability in response to purinergic modulators, suggesting that future clinical trials should differentiate subjects according to their genotype [11]. 

## 6. Purinergic Metabolism and Biomarkers 

**Figure 1:**
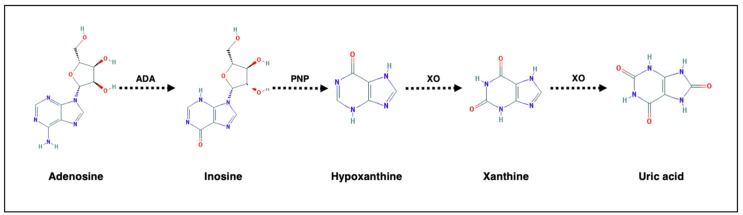
Adenosine metabolism. ADA = adenosine deaminase; PNP = Purine nucleoside phosphorylase; XO = xanthine oxidase. Chemical formulae are retrieved from PubChem (https://pubchem.ncbi.nlm.nih.gov).

Variations of adenosine metabolism have been hypothesized to be associated with major affective disorder. Adenosine, through adenosine deaminase (ADA) and xanthine oxidase (XO), is in turn metabolized to inosine, hypoxanthine, xanthine, with uric acid representing the end product of adenosine turnover (Figure 1). 

Studies in the early 1980s highlighted possible variations of adenosine metabolites, such as xanthine and hypoxanthine, in cerebrospinal fluid (CSF), showing significant correlations with both depressive symptoms [80,81] and monoamine metabolites [78]. About 20 years ago, Elgün and colleagues [82] tested the function of ADA, the enzyme responsible for the conversion of adenosine to inosine, in blood samples of 30 subjects with depression (18 with major and 12 with minor depression). The authors found a reduction of ADA activity, possibly reflecting an impaired immune state in major depressive disorder, with an inverse relationship between enzyme activity and severity of depression. However, mixed results are available in this field. More recently, Herken and colleagues [83] showed that both ADA and XO levels in subjects with major depressive disorder (*N* = 36) were higher than in healthy controls (*N* = 20). Interestingly, ADA levels further increased, whereas XO decreased, after 8 weeks of antidepressant treatment. In addition, a significant increase of XO activity in the thalamus and the putamen of patients with recurrent depression have been found [84]. Potential antidepressant actions of inosine have been shown in several preclinical studies [85,86,87,88]. Kaster and colleagues [86] showed that mice treated with inosine had higher anti-immobility effects in the forced swim and in the tail suspension tests. Inosine transiently increases its concentration in the brain enhancing neuronal proliferation [87]. Changes in the extracellular signal-regulated kinases (ERK) and cyclic AMP response element binding protein (CREB) signaling pathway in the hippocampus and prefrontal cortex were hypothesized as the target of the antidepressant action of inosine [88].

Several studies have investigated levels of adenosine metabolites, namely uric acid, in the peripheral blood of subjects with major depressive disorders. Uric acid is the end product of endogenous purine metabolism. Its production and metabolism are complex processes involving various factors that regulate hepatic production, as well as renal and gut excretion of this compound [89]. Uric acid has antioxidant effects, accounting for over half of the free radical scavenging activity, and is influenced by diet and different drugs [90]. Low levels of uric acid in CNS may impair cell antioxidant capacity. Uric acid may be a useful biomarker of the purinergic system, since central and peripheral levels may be correlated [90]. Enhanced activity of adenosine on A2A receptors may be associated with reduced adenosine turnover and lower levels of uric acid [91]. A recent systematic review and meta-analysis [92], based on 14 studies, has shown that individuals with major depressive disorders had levels of uric acid lower than healthy controls (Hedges’ g = −0.30; *p* = 0.003), as recently confirmed by recent, additional, large cohort studies [93]. Findings supported the hypothesis that uric acid levels may represent a state marker of depression, since the effect was significant only for studies including drug naïve/free individuals (Hedges’ g = −0.55; *p* = 0.023) and serum uric acid levels were significantly increased after antidepressant treatment [92]. Consistently, data from two independent cohort studies estimated that high plasma levels of uric acid were associated with antidepressant medication use [94]. Another meta-analysis has shown that uric acid levels in individuals with depression were significantly lower than in those suffering from bipolar disorder [95]. Interestingly, subjects with bipolar disorder might have increased uric acid levels [96,97] and might benefit from drugs lowering uric acid [98]. Consistent with these findings, uric acid has been proposed as a diagnostic marker that may differentiate ‘unipolar’ and bipolar depression [99]. It is noteworthy that variations of peripheral levels of uric acid have been correlated with several brain functions. A study based on functional magnetic resonance imaging (fMRI) during a psychosocial stress task showed that activity within the bilateral hippocampal complex varied with salivary uric acid levels [100], suggesting that these might modulate stress-related hippocampal activity. In addition, preliminary voxel-wise correlation analyses showed effects of uric acid on the alterations of white matter connectivity in subjects with major depressive disorder [101]. Purinergic system dysregulation in major depressive disorder has been pointed out by an observational study comparing 99 individuals with depression and 253 healthy controls [102]. Data demonstrated lower levels of both inosine and guanosine, as well as higher levels of xanthine. A recent study carried out a metabolic profiling of plasma samples to explore the potential biomarkers of major depressive disorder in children and adolescents [103]. Authors identified several abnormal pathways, including purine metabolism, and highlighted that inosine might be a possible independent diagnostic biomarker of depression, achieving an area under the receiver operating characteristic curve of 0.999 and 0.866 in the identification of drug-naïve and drug-treated subjects with major depressive disorder, respectively.

Finally, variations in purinergic metabolites were estimated in subjects treated with antidepressants. It has been shown that adenosine concentrations in plasma increased after citalopram administration in subjects with major depression [104]. More recently, a significant decrease of hypoxanthine and xanthine plasma levels after antidepressant treatment with citalopram/escitalopram was shown in 290 individuals with major depression [105]. 

Possible variations of peripheral markers of the purinergic system are summarized in Table 1.

## 7. Conclusions

Although several open questions remain, the purinergic system represents a promising research area for insights into the molecular basis of depression, characterizing a potential target for novel therapeutics [13,14,21]. Purinergic signaling may play a role in the pathophysiology of depression involving the inhibition of A1 and the activation of A2A receptors, as well as P2 receptors. In particular, A2A and P2X7 receptors have been identified as important targets for treatment of mental disorders [11,55]. Preliminary studies highlighted the possible role of purinergic system peripheral biomarkers in subjects with depression, even though underlying biological mechanisms and effects of clinical confounders or mediators should be clarified. Elucidating possible purinergic system variations may help to clarify its potentially causal nature exploring the depressive illness via a “personalized” approach. Future research should explore new approaches, such as epigenetics and proteomics, to further clarify the role of the purinergic system in affective disorders [16]. Moreover, though several preclinical studies analyzing P1 and P2 receptors are available, clinical trials are obviously needed to test the antidepressant potential of purinergic modulators in humans.

## Figures and Tables

**Table 1:** Variations of serum/plasma purinergic metabolites and enzymatic activity in depression.

Purinergic Metabolite/Enzyme	Variation
Adenosine	Increase after antidepressant treatment [99,100]
Inosine	Decrease in adults [97], children and adolescents [98] with depression
Hypoxanthine	Decrease in children and adolescents with depression [98]
Xanthine	Increase in adults with depression [97]
Uric acid	Decrease in depression, increase after antidepressant treatment [87]
Adenosine Deaminase	Decrease [78] or increase [79] in depression and after antidepressant treatment [79]
Xanthine Oxidase	Increase in depression and decrease after antidepressant treatment [79]
